# Patient-reported treatment satisfaction in essential tremor: levels of satisfaction and predictors of satisfaction

**DOI:** 10.1177/17562864241271994

**Published:** 2024-10-07

**Authors:** Anjali Varghese, Diane S. Berry, Ali Ghanem, Nora C. Hernandez, Elan D. Louis

**Affiliations:** Department of Neurology, University of Texas Southwestern Medical Center, Dallas, TX, USA; Department of Neurology, University of Texas Southwestern Medical Center, 5323 Harry Hines Blvd, Dallas, TX 75390, USA; Department of Neurology, University of Texas Southwestern Medical Center, Dallas, TX, USA; Department of Neurology, University of Texas Southwestern Medical Center, Dallas, TX, USA; Department of Neurology, University of Texas Southwestern Medical Center, Dallas, TX, USA; Peter O’Donnell Jr. Brain Institute, University of Texas Southwestern Medical Center, Dallas, TX, USA

**Keywords:** essential tremor, clinical, treatment, medication, survey

## Abstract

**Background:**

Although managing symptoms is paramount for both essential tremor (ET) patients and their healthcare providers, studies of treatment satisfaction are surprisingly lacking.

**Objectives:**

We evaluated the satisfaction of patients who used a range of treatments and assessed the relation of a wide array of factors to satisfaction.

**Methods:**

One hundred four ET participants (age = 74.5 ± 10.2 years) completed a battery of self-report items. These included demographic information, measures of tremor and clinical history, psychological state, current ET treatment, and a series of questions about satisfaction with treatment.

**Results:**

Analyses of responses to the four current treatment satisfaction questions revealed that the proportion of participants who were satisfied ranged from 35.0% to 57.3% (i.e., approximately 1/3 to 1/2); conversely, the proportion who were dissatisfied ranged from 9.2% to 37.0%. The remainder were neutral. Higher satisfaction levels were observed in participants who were included in treatment selection and who had undergone deep brain stimulation surgery, *p*’s < 0.05. Lower levels of satisfaction were found in participants with a negative psychological state, higher self-rated tremor severity, head/voice/jaw tremors, and more severe physical side effects; and who used botulinum toxin therapy, *p*’s < 0.05.

**Conclusion:**

Between 1/3 and 1/2 of patients were satisfied with their treatment, whereas up to 1/3 were dissatisfied. In this initial exploration of correlates of treatment satisfaction in ET patients, we identified a number of associations between satisfaction and clinical, psychological and treatment variables. Additional research is warranted to further explore the nature of these relations over time.

## Introduction

Essential tremor (ET) is a highly prevalent neurological disease that challenges affected individuals in their daily activities, significantly impacting quality of life.^1–4^ Although managing symptoms is paramount for both ET patients and their healthcare providers, studies of treatment satisfaction—the quality of patients’ overall experience with a particular treatment—are surprisingly lacking. Published studies have focused on *determinants of* satisfaction (efficacy of treatment, treatment side effects) or *results of* satisfaction (proportion of patients continuing or stopping their medication, the proportion who would repeat the procedure) but, to our knowledge, there have been very few studies of treatment satisfaction itself.^5^

The available data suggest that ET patients are, in general, not especially satisfied with their medical care. For example, no more than one-half of ET patients appear to experience tangible benefits from pharmaceutical treatment options, and many choose to discontinue these medications.^6–11^ As the most frequently prescribed ET medications have both limited efficacy and frequent side effects,^8,11,12^ this is not particularly surprising. Alternative interventions such as the use of botulinum toxin, deep brain stimulation surgery, and focused ultrasound thalamotomy have been employed as alternatives to orally administered pharmacotherapy in the treatment of ET, but have issues with limited efficacy and side effects as well.^5,13,14^ Yet in the literature, the issue of satisfaction per se has not really been addressed.

To address this gap in knowledge, we asked a sample of ET patients to complete a comprehensive series of questions about their satisfaction with their current ET treatment. Our first goal was to quantify the overall level of treatment satisfaction in patients with ET. To this end, our sample included a broad array of participants not limited by their specific treatment regimen. Second, we examined the relations of treatment satisfaction to a wide variety of variables. Although these did include questions about efficacy and side effects, we also assessed the relations of a number of additional demographic, clinical, psychological, and treatment variables to participants’ treatment satisfaction. We hope these data on patient satisfaction and its determinants will be of value to the ET community as well as those who are evaluating *patient-centered* perspectives on the current state of treatment.

## Methods

### Overview and study sample

By design, we enrolled ET patients from two sources between October and November 2022, with a targeted enrollment of 100. Specifically, we enrolled ET patients directly from a treatment center as well as ET cases who were not ascertained from a treatment setting. We included both as patients drawn from treatment settings may have especially high or low levels of satisfaction, thereby introducing a bias into the selection process. The goal was to enroll at an approximate 1:3 ratio of cases, with the larger balance of participants enrolled without these potential treatment setting-related biases.

First, we enrolled patients diagnosed with ET from our ambulatory clinics at the University of Texas Southwestern Medical Center (UTSW). Inclusion criteria were ET (International Classification of Diseases (ICD) 10 code G25.0), age 18 years or older, English-speaking, and having an appointment in our clinic within the next 30 days (i.e., to ensure that they were active and engaged patients). Exclusion criteria were diagnoses of Parkinson’s disease (to minimize the possibility of diagnostic misclassification due to Parkinson’s disease patients being mislabeled as ET) or neurocognitive disorder (ICD 10 codes G20, F03.90, G30.9, G31.84, R41.81; to minimize the possibility that cognitive impairment would invalidate responses to our surveys). This yielded 24 ET cases (“UTSW sample”). Second, we ascertained cases from a study of the environmental epidemiology of ET conducted from 2016 to 2021.^15^ In that study, ET cases were enrolled from the New York Tri-state area and were not enrolled from clinics or treatment centers.^15^ During that study, participants were asked how interested they would be in participating in future research on ET. Based on their response, each was assigned a score from 0 (not interested) to 10 (highly interested). We chose 100 participants with the highest scores (range = 7–10), of whom 80 participated, yielding a total sample size of 104.

Verbal informed consent was obtained from individuals who chose to participate, and they were provided with an electronic link to the questionnaire. Responses were anonymous and participants received no financial inducement for their participation.

### Explanatory variables

*Demographic variables*: Participants provided information about age, sex, race, and ethnicity, highest level of education, and current employment status.

*Clinical and Psychological Variables*: Participants indicated the number of visits they made to a neurologist during the previous 2 years, and self-rated the severity of their tremor on a four-point scale with endpoints of mild (1) and severe (4). They reported the age at which their tremor began; we calculated disease duration by subtracting age of tremor onset from current age. Participants reported whether tremor occurred in their dominant hand/arm, in their non-dominant hand/arm, and/or in their head, neck or jaw. They further indicated whether anyone in their family had tremor, and, if so, whether it had been diagnosed as ET.

We assessed psychological state via yes/no responses to the questions of whether patients (1) were satisfied with life; (2) felt their situation was hopeless; (3) were anxious; and (4) felt isolated. After reverse coding the satisfaction to life measure, we summed the number of affirmative responses to these four questions to create a “negative psychological state” index, which could range from 0 (low) to 4 (high). Finally, participants indicated whether or not they had dropped many of their activities or interests, and whether their alcohol use had increased due to their tremor.

*Treatment Variables*: Participants indicated whether their current treatment regimen included any of the following seven options: (1) beta-blockers, such as propranolol or metoprolol; (2) agents with varying degrees of gamma-aminobuytric acid (GABA)-ergic properties, such as primidone, gabapentin, or topiramate; (3) benzodiazepines, such as clonazepam, alprazolam, lorazepam, or diazepam; (4) botulinum toxin; (5) deep brain stimulation (DBS) surgery; (6) focused ultrasound thalamotomy; and/or (7) alternative therapy. They were assigned a value of 0 (not currently used) or 1 (currently used) for each.

Participants evaluated the extent of the physical (light-headedness, nausea) and mental health (cognition, mood) side effects associated with their current treatment regimen on two separate four-point scales with anchors of 1 (never/low) and 4 (yes/high). They further indicated whether they were willing to try new ET medications, they were satisfied with treatments they were using for health concerns other than ET, and their relationship with their provider influenced their perception of their ET treatment; each of these ratings was made on a five-point scale with anchors of 1 (disagree) and 5 (agree).

The following four measures assessed participants’ *sense of inclusion in the process leading to the selection of their treatment*: (1) treatment was explained well/ [I] understood how it worked before starting; (2) goals and expectations of [my] treatment [were] discussed before starting; (3) treatment was personalized to [my] specific issues; (4) [I] was part of the decision-making process in treatment selection. These statements were rated on 1 (strongly disagree) to 5 (strongly agree) scales. As these scales bore substantial relations to one another (*M* inter-correlation = +0.67) and similar relations to the outcome variables, their mean served as a composite measure of inclusion in the treatment selection process.

Finally, participants rank ordered the importance to them of four potential treatment goals. Specifically, these included (1) less tremor in activities of daily life; (2) less embarrassment/improved social life; (3) feel better about self/improve mood; and (4) career stability.

#### Satisfaction with current treatment

We surveyed the literature to identify preexisting, validated measures of treatment satisfaction. Although some do exist,^5,16,17^ we found none appropriate for a broad range of approaches used to treat ET that also sufficiently covered issues specific to ET. Thus, we developed four Likert type scales that assessed participants’ satisfaction with their current treatments. These included ratings of the extent to which their current treatment: (1) achieves the most important goal of treatment (i.e., the goal of treatment that they previously rank ordered as of greatest importance to them); (2) is working; (3) is a good thing for me; and (4) is worth the side effects. The four satisfaction ratings were each assessed on five-point response scales [(1) strongly disagree, (2) disagree, (3) neutral, (4) agree, and (5) strongly agree]; for each, greater agreement with that item yielded a higher score, indicating greater satisfaction. We further categorized participants’ responses to each item into three categories: dissatisfied (a rating of 1 or 2), neutral (a rating of 3), and satisfied (a rating or 4 or 5).

Participants also responded to the open-ended question of “What is your treatment NOT addressing?” Three independent coders assigned responses to one of the following four thematic categories: (1) ineffectiveness of treatment; (2) side effects of treatment; (3) cost of treatment; or (4) other. Initial mean level of agreement among coders’ assignments of responses to categories was 97%. Disagreements were resolved via discussion.

Some of our measures are Likert-type scales. Although some suggest that such measures should be treated as ordinal (i.e., categorical) data and analyzed via nonparametric approaches, the interpretation of such measures as interval level, and use of parametric procedures is established practice in many disciplines,^18^ and convincing conceptual and statistical rationales for this approach have been made elsewhere.^19–21^ Thus, we have elected to follow the commonly accepted practice of treating these data as continuous.

### Statistical analyses

We calculated descriptive statistics for sample demographic, clinical, and psychological characteristics, as well as for measures related to current ET treatment regimen, and satisfaction with that regimen (Table 1). *T*-tests, Mann–Whitney tests, and Pearson’s and Spearman’s correlations examined relations between case characteristics and level of treatment satisfaction.

**Table 1.** Distributions of demographic, clinical, psychological, treatment, and satisfaction variables.

Variable	Mean ∙ SD or *N* (percentage)	Possible range of score values
*Demographic variables*		
Age (years)	74.5 (10.2)	
Sex (female)	63 (60.6)	
Sample source		
UTSW sample	24 (23.1)	
Broader sample	80 (76.9)	
Race (Caucasian)	99 (95.2)	
Ethnicity (Non-Hispanic)	103 (99.0)	
Education		
Less than high school	1 (1.0)	
High school degree	16 (15.4)	
Associate’s degree	12 (11.5)	
Bachelor’s degree	37 (35.6)	
Master’s degree	20 (28.8)	
Doctoral degree	8 (7.7)	
Current Employment		
Full-time	11 (10.6)	
Part-time	5 (4.8)	
Retired	88 (84.6)	
*Clinical and psychological variables*		
Self-reported tremor severity^a^	2.43 (0.80)	1 (mild) to 4 (severe)
Age of tremor onset (years)	44.1 (19.8)	
Tremor duration^b^ (years)	30.6 (20.1)	
Number of visits to neurologist past 2 years	2.3 (2.3)	
Family history of tremor		
Absent	21 (23.6)	
Present, not identified as ET	22 (24.7)	
Present, identified as ET	46 (51.7)	
Location of Tremor^c^		
Dominant hand/arm	98 (94.2)	
Nondominant hand/arm	75 (72.1)	
Head/voice/jaw	55 (52.9)	
Increased alcohol use due to tremor^d^	12 (11.5)	
Dropped activities and interests^d^	47 (45.2)	
Negative psychological state index^e^	0.8 ± 1.1	0 (low) to 4 (high)
*Treatment variables*		
Current treatment^c^		
Beta-blocker	43 (41.3)	
GABA-ergic medication	39 (37.5)	
Benzodiazepine	10 (9.6)	
Deep brain stimulation surgery	8 (7.7)	
Botulinum toxin	5 (4.8)	
Focused ultrasound thalamotomy	4 (3.8)	
Alternative therapy	3 (2.9)	
Physical side effects from treatment^f^	2.0 ± 1.2	1 (never/low) to 4 (yes/high)
Mental health side effects from treatment^f^	1.6 ± 1.0	1 (never/low) to 4 (yes/high)
Relationship with provider affects perception of treatment^g^	3.1 ± 1.1	1 (strongly disagree) to 5 (strongly agree)
Inclusion in the treatment selection process^h^	3.7 ± 0.9	1 (strongly disagree) to 5 (strongly agree)
Satisfaction with treatments for non-ET issues^g^	3.8 ± 0.9	1 (strongly disagree) to 5 (strongly agree)
Most important goal of ET treatment^i^:		
Less tremor with activities of daily life	77 (79.4)	
Less embarrassment/improved social life	12 (13.3)	
Feel better about myself/better mood	4 (4.3)	
Career stability	2 (2.2)	
*Current treatment satisfaction variables*		
Current treatment helps achieve primary goal^j^	2.9 ± 1.3	1 (strongly disagree) to
Satisfied ([5] strongly agree/[4] agree)^k^	35 (35.0)	5 (strongly agree)
Neutral ([3] neutral)^l^	28 (28.0)	
Dissatisfied ([2] disagree/[1] strongly disagree)^m^	37 (37.0)	
Current treatment is working^g^	3.0 ± 1.2	1 (strongly disagree) to
Satisfied ([5] strongly agree/[4] agree)^k^	37 (37.0)	5 (strongly agree)
Neutral ([3] neutral)^l^	34 (34.0)	
Dissatisfied ([2] disagree/[1] strongly disagree)^m^	29 (29.0)	
Current treatment is a good thing for me^g^	3.7 ± 1.1	1 (strongly disagree) to
Satisfied ([5] strongly agree/[4] agree)^k^	55 (57.3)	5 (strongly agree)
Neutral ([3] neutral)^l^	30 (31.3)	
Dissatisfied ([2] disagree/[1] strongly disagree)^m^	11 (11.5)	
Current treatment is worth side effects^g^	3.6 ± 1.0	1 (strongly disagree) to
Satisfied ([5] strongly agree/[4] agree)^k^	50 (51.5)	5 (strongly agree)
Neutral ([3] neutral)^l^	38 (39.2)	
Dissatisfied ([2] disagree/[1] strongly disagree)^m^	9 (9.3)	
What is treatment NOT addressing?^n^		
Lack of efficacy/not working well	55 (76.4)	
Side effects	6 (8.3)	
Cost	1 (1.4)	
Other issue	1 (1.4)	
No problems with treatment	9 (12.5)	

Sample *N* = 104. Values reflect mean ± standard deviation or *n* (percent). *N*s may vary slightly for different measures due to occasional missing data.

a Measured on a four-point sale with endpoints of mild (1) and severe (4).

b Current age minus age of tremor onset.

c *N* and % of respondents who select response option. Response options are not mutually exclusive.

d Dichotomous response option (no = 0, yes = 1).

e Sum of endorsements of no/yes items reflecting a negative psychological state, including not satisfied with life, feel hopeless, feel anxious, and feel isolated. Ranges from 0 (no items endorsed) to 4 (all four items endorsed).

f Measured on a four-point scale with endpoints of never/low (1) and yes/high (4).

g Measured on a five-point scale with endpoints of strongly disagree (1) and strongly agree (5).

h Mean of four individual ratings assessed on five-point scales with endpoints of strongly disagree (1) and strongly agree (5).

i Rank ordered from most important (1) to least important (4) goal.

j Whether treatment helps achieve the goal rank ordered first in importance to respondent, as measured on a five-point scale with endpoints of strongly disagree (1) and strongly agree (5).

k Number and percentage of participants who selected either 5 (strongly agree) or 4 (agree) on the 1 (strongly disagree) through 5 (strongly agree) Likert response scale; these participants’ responses to this item were categorized as ‘satisfied’.

l Number and percentage of participants who selected 3 (neutral) on the 1 (strongly disagree) through 5 (strongly agree) Likert response scale; these participants’ responses to this item were categorized as ‘neutral’.

m Number and percentage of participants who selected 2 (disagree) or 1 (strongly disagree) on the 1 (strongly disagree) through 5 (strongly agree) Likert response scale; these participants’ responses to this item were categorized as ‘dissatisfied’.

n *N* (percent) of 72 participants’ responses corresponding to each of four categories after patients reporting no current treatment (13), providing a nonresponsive answer (2), or not responding to question (17) were eliminated from analysis.

Next, we calculated a series of bivariate equations in which each of the four primary satisfaction measures previously described was regressed on an individual demographic, clinical, psychological, or current treatment variable (Table 2).

**Table 2.** Bivariate linear regression equations predicting measures of patient satisfaction from demographic, clinical/psychological, and treatment use variables.

Explanatory variable	Measure of participant satisfaction			
	Achieves goal	Is working	Is good thing	Worth side effects
*Demographic variables*				
Age^a^	−0.12	−0.16	0.01	−0.06
Sex^b^	0.10	−0.04	0.05	−0.07
Education^a^	0.11	0.09	0.01	0.04
Employment^a^	−0.20	−0.17	−0.07	−0.08
Sample source^c^	0.03	0.04	−0.10	−0.07
*Clinical variables*				
Self-rated tremor severity^a^	−0.25**	−0.17	−0.15	−0.14
Age of tremor onset^a^	−0.13	−0.17	−0.09	−0.15
Tremor duration^a^	0.09	0.10	0.10	0.10
*N* neurologist visits last 2 years^a^	−0.11	−0.04	−0.10	−0.10
Family history of tremor^d^	0.01	−0.08	−0.08	−0.05
Family history of ET^d^	0.01	−0.04	−0.15	−0.03
Dominant hand/arm tremor^e^	−0.05	0.01	−0.20	−0.06
Nondominant hand/arm tremor^e^	0.03	−0.10	0.05	0.17
Head/voice/jaw tremor^e^	−0.19	−0.27**	−0.24*	−0.17
*Psychological variables*				
Increase alcohol use due to tremor^f^	−0.07	−0.04	0.01	0.08
Dropped activities/interests^f^	−0.20*	−0.19	−0.03	−0.01
Negative psychological state index^a^	−0.42***	–0.33**	–0.24*	−0.18
*Treatment variables*				
Beta-blocker use^g^	−0.19	−0.19	−0.02	0.04
GABA-ergic medication use^g^	−0.10	−0.06	0.10	0.16
Benzodiazepine use^g^	−0.11	−0.01	−0.05	0.02
Botulinum toxin use^g^	−0.16	−0.01	−0.22*	−0.09
Deep brain stimulation surgery^g^	0.28**	0.31**	0.26**	0.21*
Focused ultrasound thalamotomy^g^	−0.07	0.04	−0.17	0.04
Alternative therapy use^g^	−0.03	0.05	0.05	−0.04
Side effects, physical^a^	−0.25**	−0.27**	−0.23*	−0.20*
Side effects, mental health^a^	−0.33***	−0.33***	−0.33***	−0.28**
Relationship w/provider affects perceptions of treatment^a^	−0.01	0.03	−0.04	−0.17
Inclusion in the treatment selection process^a^	0.42***	0.46***	0.43***	0.47***

Sample *N* = 104. Values = standardized beta coefficient; *p*’s ⩽ 0.05 appear in bold; *p ⩽ 0.05; **p ⩽ 0.01; ***p ⩽ 0.0001.

a Higher values indicate greater levels of the described construct.

b 1 = male, 2 = female.

c 1 = research sample, 2 = UTSW sample.

d 0 = history absent, 1 = history present.

e 0 = tremor absent, 1 = tremor present.

f 0 = no, 1 = yes.

g 0 = treatment not currently in use, 1 = treatment currently in use.

Finally, we calculated four multivariate regression equations examining the independent association of demographic, clinical, psychological, and treatment variables to each of the four satisfaction measures. Specifically, all variables significantly associated with a given measure of satisfaction (at *p* ⩽ 0.05) in the previously described bivariate regressions (Table 2) were simultaneously included as independent variables in the associated multivariate model (Table 3).

**Table 3.** Multivariate linear regression equations predicting treatment satisfaction from demographic, clinical, and treatment use variables.

Outcome	Explanatory variable	*B*	*p*
Current treatment helps achieve primary goal^1^			
	Tremor severity^a^	−**0.30**	**0.002**
	Dropped activities/interests^b^	0.01	0.99
	Negative psychological state^a^	−**0.23**	**0.02**
	Deep brain stimulation surgery^c^	**0.31**	**0.001**
	Physical side effects^a^	−0.19	0.11
	Mental health side effects^a^	−0.02	0.90
	Inclusion in the treatment selection process^a^	**0.32**	**0.001**
Current treatment is working^2^			
	Head/voice/jaw tremor present^d^	**–0.19**	**0.02**
	Negative psychological state^a^	**–0.18**	**0.04**
	Deep brain stimulation surgery^c^	**0.21**	**0.04**
	Physical side effects^a^	**–0.24**	**0.02**
	Mental health side effects^a^	0.01	0.95
	Inclusion in the treatment selection process^a^	**0.38**	**0.001**
Current treatment is a good thing for me^3^			
	Head/voice/jaw tremor present^d^	−0.16	0.09
	Negative psychological state^a^	−0.10	0.30
	Botulinum toxin use^c^	**–0.21**	**0.02**
	Deep brain stimulation surgery^c^	0.12	0.21
	Physical side effects^a^	−0.12	0.32
	Mental health side effects^a^	−0.08	0.54
	Inclusion in the treatment selection process^a^	**0.39**	**0.001**
Current treatment is worth side effects^4^			
	Deep brain stimulation surgery^c^	0.10	0.29
	Physical side effects^a^	−0.20	0.13
	Mental health side effects^a^	−0.08	0.55
	Inclusion in the treatment selection process^a^	**0.44**	**0.001**

DF vary slightly due to missing data. *B* = standardized beta. Bolded *p* values are significant at *p* < 0.05. High scores on the outcome variable suggest treatment is perceived more positively than do low scores.

a Higher values indicate greater levels of the described construct.

b 0 = no, 1 = yes.

c 0 = treatment not currently in use, 1 = treatment currently in use.

d 0 = tremor absent, 1 = tremor present.

^1^*F*_equation_ (7, 96) = 10.02, adjusted *R*^2^ = 0.40, *p* < 0.001.

^2^*F*_equation_ (6, 96) = 10.61, adjusted *R*^2^ = 0.38, *p* < 0.001.

^3^*F*_equation_ (7, 95) = 7.08, adjusted *R*^2^ = 0.31, *p* < 0.001.

^4^*F*_equation_ (4, 95) = 9.34, adjusted *R*^2^ = 0.26, *p* < 0.001.

## Results

### Case characteristics: Demographic, clinical, and current treatment measures

The distributions of demographic, clinical, psychological, and current treatment and satisfaction measures for the 104 participants appear in Table 1. Beta-blockers and GABA-ergic medications were the most commonly reported current specific treatments for ET (41.3% and 37.5%, respectively). The most frequently selected primary goal of ET treatment was to experience less tremor during daily activities (*n* = 77, 79.4%). The mean frequency of side effects participants associated with their current treatment (1 = never/low to 4 = yes/high) were 2.0 ± 1.2 and 1.6 ± 1.0 for physical and mental health side effects, respectively.

### Overall satisfaction with current ET treatment

The mean of participants’ satisfaction with the extent to which their current treatment achieved its primary goal, “is working,” “is a good thing for me,” and “is worth the side effects” were 2.9, 3.0, 3.7, and 3.6, respectively (see Table 1) on the 1 to 5 scale. All four items displayed reasonable variability (SD range = 1.0–1.3), and responses to each of the four items fully spanned scale values (range for each = 1–5). None of the four satisfaction variables differed significantly as a function of participant source (i.e., UTSW sample vs broader research participant sample) (all *p*’s > 0.35), sex (all *p*’s > 0.32), age (all *p*’s > 0.10), or education (all *p*’s > 0.28).

For each of the four current treatment satisfaction questions, we categorized participants’ response to each item as either “dissatisfied” (a rating or 1 or 2), “neutral” (3), or “satisfied” (4 or 5). The proportion of participants who were satisfied ranged from 35.0% to 57.3% (i.e., approximately 1/3 to 1/2); conversely, the proportion who were dissatisfied ranged from 9.3% to 37.0%. The remainder were neutral.

We performed content analyses of answers to the open-ended question of what issue participants’ current treatment regimen did not address. Of our 104 participants, 72 provided usable responses to this question (Table 1). Only nine (12.5%) of the 72 respondents indicated no problem or concern with their current treatment, suggesting few were fully satisfied with their treatment. Consistent with previous work,^22^ the most commonly cited specific participant complaint was treatment ineffectiveness (*n* = 55, 76.4%).

### Predictors of treatment satisfaction

#### Current treatment helps achieve primary goal of treatment

Separate bivariate linear regressions revealed significant associations between participants ’ ratings of whether their current treatment helped them achieve their primary goal of treatment and seven individual variables (Table 2). We then computed a multivariate linear regression equation in which these seven measures were simultaneously entered as explanatory variables and participants ’ ratings of primary treatment goal achievement served as the outcome measure (Table 3). This equation revealed four significant independent predictors of participants ’ ratings. Specifically, lower tremor severity, *B* = −0.30, *p* = 0.002, greater inclusion in the treatment selection process, *B* = 0.32, *p* < 0.001, a less negative psychological state, *B* = −0.23, *p* = 0.02, and the use of DBS surgery, *B* = 0.31, *p* = 0.001, were significantly associated with participants’ perception that their current treatment regimen was helping them achieve their primary treatment goal (Values listed reflect standardized betas.)

#### Current treatment is working

Bivariate analyses revealed a pattern of associations between participants’ ratings of whether their current treatment is working and six independent variables (Table 2). The multivariate model (Table 3) that included these six factors as explanatory variables revealed that participants who did not experience head, voice, or jaw tremor, *B* = −0.19, who were more included in their treatment selection process, *B* = 0.38, who reported a less negative psychological state, *B* = –0.18, who had undergone DBS surgery, *B* = 0.21, and who reported fewer physical side effects, *B* = −0.24, reported that their treatment was working significantly better than did other patients, all *p*’s < 0.04.

#### Current treatment is a “Good Thing for Me.”

Bivariate regressions revealed significant associations between participants’ ratings of whether their current treatment “is a good thing for me” and seven variables (Table 2). The associated multivariate equation revealed that use of botulinum toxin to treat ET was significantly and negatively associated with this outcome, *B* = −0.21, *p* ⩽ 0.02. Ratings of the extent to which participants felt included in the treatment selection process were significantly and positively associated with the extent to which treatment was judged to be “a good thing”, *B* = 0.39, *p* ⩽ 0.001 (Table 3).

#### Current treatment is “worth the side effects.”

Finally, bivariate regressions revealed significant associations between participants’ ratings of whether their current treatment is “worth the side effects” and four independent variables (Table 2). The accompanying multivariate equation revealed that the extent to which participants’ felt included in treatment selection was the only variable significantly associated with this measure of satisfaction, *B* = 0.44, *p* ⩽ 0.001 (Table 3).

## Discussion

Our primary goals were (1) to assess participants’ satisfaction with their current treatment for ET; and (2) to identify factors associated with treatment satisfaction. Among the four current treatment satisfaction questions, the proportion of participants who were satisfied ranged from 35.0% to 57.3% (i.e., approximately 1/3 to 1/2); conversely, the proportion who were dissatisfied ranged from 9.2% to 37.0%. The remainder were neutral. Although treatment satisfaction was unrelated to demographic variables such as age, sex, or education, analyses revealed associations between greater participant satisfaction and (1) low tremor severity; (2) lack of tremor in the head, voice, or jaw, (3) feelings of inclusion in treatment selection, (4) being in a negative psychological state, (5) fewer physical side effects, (6) use of DBS, and (7) nonuse of botulinum toxin.

The extent to which participants felt they had been included in and part of the treatment selection process bore the strongest relation of any explanatory variable to each of the four satisfaction items. That a sense of inclusion and influence over treatment yields positive patient outcomes is not surprising, given the large psychological literature on the health benefits of both perceived and actual control over health related decisions.^22–24^

Participants who indicated that they had undergone DBS surgery for ET also provided higher ratings of the extent to which their treatment achieved its primary goal and was working than did participants who had not had DBS surgery. These data are generally consistent with previous work that reveals reasonably high levels of general satisfaction with DBS.^25–27^

Botulinum toxin use was related to one outcome measure; people who did *not* receive this treatment provided higher ratings of the extent to which their treatment was a good thing than did those who did. This treatment has been reported to yield better results with the treatment of cranial (head and voice) tremors than limb tremors.^12,13,28^ Thus, if our data were drawn from a sample of botulinum toxin users who did not exhibit cranial tremor, that might account for the comparatively lower levels of satisfaction we observed in participants receiving this treatment. However, 80% of our participants who used botulinum toxin did, in fact, exhibit cranial tremor; if anything, this should bias our sample toward experiencing *greater* treatment satisfaction.

Participants who reported less-severe tremor also felt their treatment better achieved its primary goal than did other participants. As the vast majority of participants (80%) identified “less tremor in activities of daily life” as their primary treatment goal, its relation with tremor severity is not surprising. Moreover, the link of tremor severity and dissatisfaction is consistent with the report that ET patients with greater tremor also report more negative social consequences of that tremor.^29^ Participants with more negative psychological states were less satisfied than others with the extent to which their treatment achieved its primary goal. This negative halo effect in ET patients is consistent with previous data;^29^ a negative self-view on one specific dimension tends to correlate with evaluations on other dimensions (i.e., feeling worse can lead to less-favorable evaluation of one’s therapeutic benefits). Finally, fewer physical side effects and the absence of head, voice, or jaw tremor were associated with higher participant ratings of how well their treatment was working. The link between cranial tremor and satisfaction likely reflects the fact that head tremor tends to be less responsive than limb tremor to many ET treatments.^30^

The lack of association between several explanatory variables and satisfaction is also noteworthy. First, no measured category of pharmaceutical treatment for ET was significantly related to participant satisfaction. This is significant, as beta-blockers, GABA-ergic medications, and benzodiazepines are among the most frequently prescribed treatments for ET.^7,9^ Moreover, these were the three most commonly reported treatments among our participants. Second, although assessments of efficacy and side effects dominate the current literature on treatment satisfaction, neither emerged as the primary determinant of satisfaction in the current data. Although analyses of the free response data did reveal that efficacy was the most frequently cited specific concern about treatment, our measure most related to efficacy (self-rated tremor severity) emerged as a significant explanatory variable for only one of the four satisfaction measures. Moreover, participants’ reports of the severity of mental health side effects did not predict any satisfaction measures, and reports of the severity of physical side effects predicted only one measure of satisfaction. In addition, a majority of participants actually felt their treatment was worth whatever side effects they experienced. Thus, although measures of efficacy and side effects are clearly related to satisfaction, these concepts are not interchangeable. As a result, the existing literature likely provides a limited view of what contributes to ET patients’ satisfaction.

There are limitations to the current study. First, our analyses capture only one moment in time in our patients’ experience, making it difficult to draw causal inferences about any associations we observed. Follow-up studies in which patients are assessed immediately before starting a new treatment and followed across time are needed to shed light on these issues, as well as to rule out potential alternative explanations of observed links. On a related note, future studies would benefit from assessing the point in treatment at which patients are observed (e.g., beginning of treatment, after a year of treatment). This is important, as the nature of the relations among these variables likely shift over time. Similarly, ET patients often have a history of multiple treatment trials over time, so that a dynamic view of satisfaction is preferable. Again, this can best be captured in a longitudinal design. Second, our satisfaction measures involve Likert-type scales developed specifically for this study. It would, of course, have been preferable to use established scales, but no validated assessment meeting our needs was available. Third, although it is difficult to imagine a valid method of measuring satisfaction that does not involve self-report, it would be desirable to obtain more objective assessments of other variables. For example, future work exploring the relation observed between tremor severity and satisfaction could include expert rater assessments of tremor on measurement scales such as the Tremor Research Group Essential Tremor Assessment Scale (TETRAS)^31^ or the Washington Heights-Inwood Genetic Study of Essential Tremor rating scale (WHIGET).^32,33^ Fourth, our sample was largely Caucasian, and well-educated. It would be desirable to replicate these findings with more diverse patient samples. Fifth, some participants had consented to be contacted for involvement in future research, possibility influencing the generalizability of the data. Finally, the number of participants who underwent some individual treatments (e.g., botulinum toxin, surgical treatments) was relatively small, which may limit the predictive power of these variables. More specifically, a low number of participants taking part in a given treatment increases the likelihood of a nonrepresentative sample of users of that treatment yielding spurious results. In addition, small treatment subgroups preclude the exploration of potential moderator variables that could affect the association between a given treatment and satisfaction.

Our work augments the current literature on treatment satisfaction in several important ways. First, to our knowledge, there have been virtually no studies of treatment satisfaction itself. Second, use of a specific type of ET surgical treatment or medication was not a requirement for participation. Thus, our participants represent a broader and more heterogeneous group of ET patients than is typically the case in studies of patients using one treatment modality (e.g., DBS surgery). Third, by design, we sampled patients from two sources, with the majority of participants enrolled without potential inherent treatment setting-related biases. Interestingly, in this sample analyses revealed no differences in treatment satisfaction between these two groups of participants. Fourth, with great intention, we designed the study to obtain data on a wide array of variables as potential predictors of treatment satisfaction. This expanded focus reveals some intriguing associations that warrant future attention. For example, given the well-documented health consequences of patients’ sense of control over their health decisions,^23,34^ the reported association between patient’s feelings of involvement in treatment decisions and satisfaction seems an especially promising area for future study. We hope these data on patient satisfaction and its determinants will be of value to the ET community as well as those who are evaluating *patient-centered* perspectives about the current state of treatment.

## Declarations

**Ethics approval and consent to participate:** The study was approved by the University of Texas Southwestern Medical Center Institutional Review Board, approval number = STU-2022-0113. Participants provided verbal informed consent when initially recruited for participation. Verbal informed consent was used because contact with participants was typically made by telephone. The use of verbal consent was approved by the University of Texas Southwestern Medical Center Institutional Review Board.

**Consent for publication:** Not applicable.

**Availability of data and materials:** Data may be obtained by contacting EDL.
